# Colic

**Published:** 1890-05

**Authors:** 


### COLIC.

Roughly speaking, colic is due to irregular, violent, and usually very painful contractions of the great intestine. The causes of this complaint are very numerous. In some cases the disease is due to constipation and consequent distention of the colon. Sometimes over fatigue will bring it on ; occasionally eating some food which does not agree with the constitution. Many positively awful cases in which the sufferer longed for death as a relief from his agony, have been due, in my experience, to such trifling causes as eating a bit of plum cake or plum pudding, a few cherries, currants, gooseberries, grapes, an unripe apple or pear. Water contaminated with lead or some other metal is one of the most frequent of all causes, one form, painters' colic, having gained a very unenviable notoriety. Exposure to cold is a frequent cause, while many sufferers know, to their cost, that the slightest anxiety or overwork will give them a sharp attack. Middle-aged and elderly women of rather full habit, are more liable to colic than men. Careful regimen is valuable as far as it goes, but is very far from being all-sufficient, for in many cases the keenest scrutiny will not detect any errors of diet, although worry, overwork, and cold, combined with constitutional predisposition, account for most cases.

